# Marked disability and high use of nonsteroidal antiinflammatory drugs associated with knee osteoarthritis in rural China: a cross-sectional population-based survey

**DOI:** 10.1186/ar3212

**Published:** 2010-12-29

**Authors:** Jianhao Lin, Marlene Fransen, Xiaozheng Kang, Hu Li, Yan Ke, Zhiqiang Wang, Yuqing Zhang, Steve Su

**Affiliations:** 1 Arthritis Centre, Peking University People's Hospital, South Street Xizhimen, Beijing 100044, PR China; 2 Faculty of Health Sciences, University of Sydney, East Street, Lidcombe 1825, Australia; 3 Peking University School of Oncology, Beijing Cancer Hospital and Institute, Fucheng Road, Beijing 100142, PR China; 4 Clinical Epidemiology Research and Training Unit, Boston University School of Medicine, Albany Street, Boston, MA 02118, USA; 5 School of Mathematics and Statistics, University of Western Australia, Stirling Highway, Crawley 6009, Australia

## Abstract

**Introduction:**

The burden of disability, analgesia, and health services use associated with knee pain and osteoarthritis (OA) in developing countries is relatively unknown, despite a high proportion of these populations required to be engaged in heavy occupational physical activity throughout their life span. The aim of this survey was to estimate the burden of disability, analgesia, and health services use associated with knee pain in rural China.

**Methods:**

This was a population-based cross-sectional survey among residents, aged 50 years and older, of Wuchuan County, Inner Mongolia. Participants completed an interviewer-based questionnaire, evaluating knee pain and associated disability, analgesia, and health services use, and obtained bilateral standardized weight-bearing knee radiographs.

**Results:**

Of the 1,027 participants, 513 (50%) reported knee pain on most days of at least 1 month in the past year, with 109 (21%) also demonstrating radiographic OA (Kellgren-Lawrence grade ≥2) in the symptomatic knee. Adjusting for age, gender, body mass index (BMI), education, and back pain, the presence of knee pain was associated with significantly greater difficulty in walking, climbing 10 steps, stooping, completing cleaning chores, and preparing meals. Among the 513 subjects with knee pain, the additional presence of radiographic evidence of OA was significantly associated with more occasions of "unbearable" pain (59% versus 36%) and restricted activity (64% versus 39%), as well as increased use of nonsteroidal antiinflammatory drugs (NSAIDs) (88% versus 78%) and the reported number of doctor visits (59% versus 33%) in the past year. The use of paracetamol for knee pain was rare (6% versus 2%).

**Conclusions:**

Knee pain is highly prevalent in rural northern China. The associated significant disability and marked preferential use of NSAIDs as analgesia should be of concern in these communities reliant on heavy occupational physical activity for their livelihood. The findings will be useful to guide the distribution of future health care resources and preventive strategies. A similar article has been published in the Chinese language journal, *National Medical Journal of China*.

## Introduction

Knee pain due to osteoarthritis (OA) is considered a highly prevalent disease among older persons [1-4]. However, most large population-based observational studies evaluating OA disease prevalence have been conducted in North America or Europe although it has been estimated that by 2050, almost four fifths of the world's older population (65 years and older) will be living in less-developed regions of the world [5]. To start to address this disparity, the WHO-APLAR COPCORD collaboration conducted a series of observational studies evaluating the prevalence of rheumatologic diseases in several Asian populations [6-12]. However, whereas these studies provided estimates of region-specific OA disease prevalence, these could not be compared with prevalence reported from North America or Europe because of the lack of standardization in disease definition. Further, these studies provided little information on associated disability or medication and health services use.

A recent population-based survey conducted in a rural area in Northern China (Wuchuan, Inner Mongolia) [13] and using a standardized definition of knee pain and knee radiographs provided initial evidence that knee OA prevalence or disease presentation observed in North America or Europe may not be directly extrapolated to rural communities in less-developed regions of the world. The prevalence of symptomatic knee OA in Wuchuan County was significantly higher compared with age- and gender-compatible peers in Framingham, Massachusetts, North America [13]. Further, although the prevalence of radiographic knee OA found in rural Wuchuan County was similar to that demonstrated in Beijing, the prevalence of symptomatic knee OA was significantly higher in the rural compared with the urban community [13].

Although simple region-specific disease-prevalence estimates are important, associated symptom severity (pain, disability) and use of treatments and health services will determine the actual disease burden. The individual OA disease burden demonstrated in high-income countries or among urban cohorts with mostly sedentary occupations cannot be directly extrapolated to less-developed countries or rural communities. Region-specific information will be required to guide the distribution of future health care resources and preventive strategies.

The aim of this study was to describe and compare levels of pain, physical disability, and use of medications and health services among people with knee pain and symptomatic knee OA with those of their unaffected peers among older people living in Wuchuan County, a rural region in Northern China.

## Materials and methods

The sampling methods of this survey among people aged 50 years and older living in Wuchuan County, Inner Mongolia, have been detailed elsewhere [13]. In brief, within five randomly selected communities, a compact segment-sampling method was used to identify clusters, each containing six to eight villages. Clusters were then selected with a probability proportional to the population size at the last census. Then a sketch map was drawn of each selected cluster (total, 30), showing the dwellings present. The selected clusters were each split into a small number of segments (two to four segments), such that the number of dwellings per segment was always roughly the same (20 to 30 households). One segment was then chosen at random from each cluster, and all households in the segment were included in the survey. Specific ethnic groups were neither targeted nor excluded. Ethnicity was further not recorded in this survey, as the cohort was almost exclusively Han Chinese (> 99%).

The study was approved by the Peking University Health Science Center Ethics Committee, and informed consent was obtained from all study participants according to the Declaration of Helsinki.

### Participants

Trained health professionals administered the survey questionnaires, as it was anticipated that many study participants would be illiterate. All interviewers, clinical examiners, and x-ray technicians were trained under the supervision of the study chief investigators (XK, JL). Trained interviewers went door-to-door to enumerate and interview all men and women, aged 50 years or older, within the selected households who were self-described residents of Wuchuan County. Individuals who self-reported rheumatoid arthritis, cerebrovascular disease, or a history of lower-limb surgery were excluded from further participation, as it would be difficult to isolate the pain and disability burden due to knee osteoarthritis from that attributable to cerebrovascular disease, rheumatoid arthritis, or prior lower-limb surgery.

Subjects were interviewed at their homes or work places. At the end of the interview, all study participants were invited to one central examination site at Wuchuan Hospital for a clinical examination and knee radiographs on the same day. Transportation to the hospital was provided.

After the collection of basic demographic data, all survey participants were asked to respond to the Medical Outcome Study Short Form (SF-12) standard questionnaire (validated Chinese language version) evaluating health-related quality of life. The SF-12 questionnaire is well validated for use among patients with OA [14]. To increase precision and reduce the number of statistical comparisons needed, algorithms were developed from the eight health domains assessed, to calculate two summary measures: the Physical Component Summary Scale Score (PCS) and the Mental Component Summary Scale Score (MCS) [14].

Survey participants further reported the level of difficulty (that is, no difficulty, some difficulty, much difficulty, unable to do, don't know, or don't do) when performing the following specific activities: walking for two li (approximately 1 kilometer); walking up 10 steps without resting, stooping, crouching, or kneeling; cleaning chores around the house like folding quilts, sweeping, dusting, or straightening up; or preparing meals.

Subjects who reported having had pain, aching, or stiffness lasting at least a month in or around the knee in the past 12 months were further asked (In the past 12 months): How severe was the pain usually? (usually bearable, sometimes unbearable, mostly or always unbearable); Have you limited your daily activities, such as required by your job or housework, because of pain, aching, or stiffness in your knee? (No, Yes).

Subjects who reported knee pain were asked if they had received any of the following treatments in the past 12 months for their knee pain: herbal medicine, acupuncture, massage, other traditional Chinese medicine, nonsteroidal antiinflammatory drugs (including diclofenac (voltaren), fenbid, ibuprofen, sulindac (clinoril), naproxen (naprosyn), indomethacin suppository), paracetamol/acetaminophen/Tylenol, physiotherapy, or surgery. These participants were also asked if they had seen a doctor in the past 12 months for knee pain, aching, stiffness, or arthritis.

### Clinical examination and knee radiograph

Height was measured with a wall-mounted stadiometer, by using the average of two measurements taken. Body weight was assessed by using a balance-beam scale with 0.1-kg precision. A posterior-anterior weight-bearing semiflexed radiograph was taken of both knees strictly according to a validated acquisition protocol [15]. Radiographs were read by the study chief investigator (XK), and Kellgren-Lawrence grades (0 to4) were assigned. The reading methods of this survey have been detailed elsewhere [13].

Knee pain was defined as having a positive response to the question, "In the past 12 months, have you had knee pain lasting most days of at least a month?" Symptomatic knee OA was defined as having knee pain and scoring a Kellgren-Lawrence grade ≥2 in the radiograph of this knee.

### Statistical analysis

We divided participants into three groups:

1) subjects with no knee pain in the past 12 months;

2) subjects with knee pain in at least one knee in the past 12 months, but without radiographic OA (Kellgren-Lawrence grade <2) in a symptomatic knee; and

3) subjects with knee pain in at least one knee in the past 12 months and with radiographic OA (Kellgren-Lawrence grade ≥2) in the symptomatic knee.

By using an analysis of variance for continuous variables and a χ^2 ^test for categoric variables, we compared sociodemographic characteristics of the three groups of participants. We examined the relation of knee pain and symptomatic knee OA to the prevalence of various measures of physical disability with multivariable logistic regression models. In the regression models, we adjusted for age, gender, BMI, presence of back pain, and years of education. Among those with knee pain, we evaluated the association of radiographic knee OA and use of health services and medications by using the multivariable logistic regression model. All the analyses were performed by using R-2.6.1, a statistical program developed by the R Foundation for Statistical Computing, Vienna, Austria [16].

## Results

**Figure 1:**
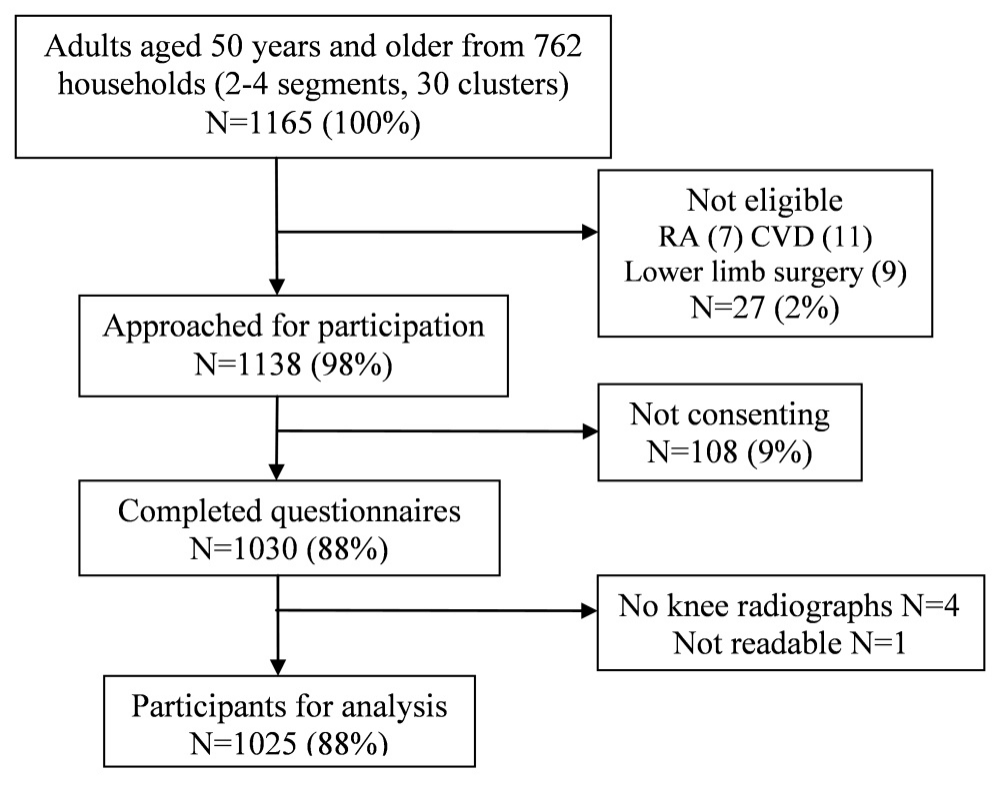
**Wuchuan County Osteoarthritis Study**. Recruitment.

In total, 1,165 individuals aged 50 years and older were identified in the randomly selected households in Wuchuan County, Inner Mongolia (Figure 1).

### Wuchuan OA Study

#### Sample population and exclusions

Of these, 27 subjects were excluded from further study participation (rheumatoid arthritis (*n *= 7), cerebrovascular disease (*n *= 11), or a history of lower-limb surgery (*n *= 9)). Of the remaining 1,138 residents approached, 1,030 (91%) consented to participate in the study and completed the home interview during the months of October and November 2005. People declining to consent were mostly older compared with the study participants (mean (SD) 64 (7) versus 58 (8) years, respectively). Only three consenting participants did not attend the radiographic examination.

Of the 1,027 participants with knee radiographs, 513 (50%) reported having knee pain in the past 12 months. Of these participants with knee pain, 109 (21%) had radiographic disease (Kellgren-Lawrence grade ≥2) in the symptomatic knee. At the time of data analysis, it was discovered that 24 men and 26 women were actually younger than 50 years (48 or 49 years), and two participants had a history of minor knee surgery. As the age difference or surgery was minimal, it was decided to retain these data in the analysis. The mean body mass index (BMI) of all survey participants was 22 kg/m^2^, with only 63 (6%) considered to be obese (BMI ≥28) and 219 (21%) considered overweight (BMI ≥24, but less than 28) according to specific Asian population criteria [17].

The characteristics of the three groups of participants are presented in Table 1. Significant differences were found in age, gender, BMI, presence of back pain, and years of education between the three groups. Participants with symptomatic knee OA (defined as the combination of knee pain and radiographic OA in the symptomatic knee) were slightly older, had a higher mean BMI, and were more likely to be women, compared with those without radiographic disease. The prevalence of back pain was higher in participants with knee pain (without or without radiographic disease) compared with participants without knee pain, but similar between the two categories of participants with knee pain. The majority of participants received less than 7 years of formal education. Almost all participants were farmers or had been engaged in farming as their main occupation, and most participants were still working. No significant differences were noted in the SF-12 PCS between the three groups, but persons with knee OA had a small but significantly higher SF-12 MCS compared with those without knee pain.

**Table 1:** Characteristics of the Wuchuan County OA Study participants

	No knee pain (*n *= 514)	Knee pain (*n *= 404)	Symptomatic knee OA^a^ (*n *= 109)	*P*
Age, years; mean (SD)	57.3 (7.8)	56.2 (7.4)	62.7 (8.9)	< 0.001
Female	45%	54%	68%	< 0.001
BMI (kg/m^2^), mean (SD)	22.0 (3.1)	22.5 (3.1)	24.0 (4.0)	< 0.001
Back pain	29%	51%	43%	< 0.001
< 7 years' education	74%	72%	84%	0.04
Occupation, farming	93%	90%	83%	0.002
Still working	86%	89%	67%	< 0.001
SF-12 PCS, mean (SD)	49.9 (6.2)	49.9 (6.8)	49.3 (6.8)	0.7
SF-12 MCS, mean (SD)	52.3 (6.1)	53.4 (5.7)	54.6 (7.1)	0.004

**^a^**Radiographic OA in a painful knee. MCS, Mental Component Summary Score; PCS, Physical Component Summary Score.

The results presented in Table 2 demonstrate that participants with knee pain were 2 to 4 times more likely to experience difficulty with usual daily activities compared with participants without knee pain, even when the analysis was adjusted for age, gender, BMI, presence of back pain, and years of formal education. These difficulties were even more likely to be present in established radiographic knee OA. The increased odds among people with symptomatic knee OA, compared with participants with knee pain only, was significant for walking for 2 li and "stooping, crouching, or kneeling."

**Table 2:** Self-reported physical disability

Outcome	*n*	"With difficulty"^a^	OR (95% CI) [*P*]^b^
Walk for 2 li (1 km)			
No knee pain	514	22%	1
Knee pain	402	37%	1.9 (1.4 to 2.7)
Knee OA	109	60%	3.2 (2.0 to 5.2)
			[*P *= 0.04]
Up 10 steps without resting			
No knee pain	491	30%	1
Knee pain	387	64%	4.0 (3.0 to 5.5)
Knee OA	104	78%	5.5 (3.3 to 9.8)
			[*P *= 0.27]
Stooping, crouching, kneeling			
No knee pain	514	33%	1
Knee pain	404	70%	4.2 (3.1 to 5.8)
Knee OA	109	90%	14.5 (7.5 to 31.0)
			[*P *< 0.001]
House-cleaning chores			
No knee pain	514	7%	1
Knee pain	404	14%	1.8 (1.1 to 2.8)
Knee OA	109	25%	2.2 (1.2 to 4.0)
			[*P *= 0.48]
Preparing meals			
No knee pain	449	5%	1
Knee pain	368	11%	2.2 (1.3 to 4.0)
Knee OA	102	20%	2.5 (1.2 to 5.1)
			[*P *= 0.70]

PCS, Physical Component Summary Score, adjusted for age, gender, BMI, self-reported back pain and years of education. **^a^**Reporting "some difficulty," "much difficulty," or "unable to do." ^b^[*P*] Difference knee pain versus knee OA.

A 99% to 100% response rate occurred to each of the questions regarding medications and health services use in the past 12 months (Table 3), apart from the question regarding the use of "other Western medicine," to which 11% and 23% of participants with knee pain and knee OA, respectively, responded with a "don't know." This response was removed from the analysis. Only two participants reported having had knee surgery in the past 12 months, one from each participant subgroup.

**Table 3:** Pain, activity restriction, use of health services, and medications for knee pain

	Knee pain *n *= 404	Symptomatic knee OA *n *= 109	OR (95% CI)
Pain unbearable at times	36%	59%	2.0 (1.3 to 3.3)
Limited activity	39%	64%	2.4 (1.5 to 4.0)
Herbal medicine	25%	60%	4.4 (2.7 to 7.5)
Acupuncture	20%	40%	2.2 (1.3 to 3.8)
Massage	4%	6%	1.2 (0.4 to 3.3)
Other TCM	26%	35%	1.6 (0.9 to 2.6)
NSAIDs	78%	88%	2.1 (1.1 to 4.4)
Paracetamol	2%	6%	3.4 (1.0 to 11.2)
Other Western medicine	13%	21%	2.0 (1.0 to 4.0)
Physiotherapy	10%	17%	1.1 (0.5 to 2.2)
Doctor past year?	33%	59%	2.9 (1.8 to 4.8)

Prevalence (%), adjusted odds ratio (OR), and 95% confidence interval (CI). Adjusted for age, gender, BMI, self-reported back pain, and years of education. TCM, traditional Chinese medicine.

Among those reporting knee pain, the majority (80%) had used NSAIDs for their knee pain in the past 12 months, whereas only a very small proportion had used paracetamol (3%) (Table 3). A significantly larger proportion of subjects with symptomatic knee OA, compared with those subjects with knee pain (but without defined radiographic OA), had episodes of unbearable pain, reported limited activity due to knee pain, taken NSAIDs for knee pain, accessed herbal medications, or visited a doctor in the past 12 months, with significant odds ratios ranging from 2.0 to 4.4 (Table 3).

## Discussion

The results of this population-based survey demonstrate that knee pain, with or without the presence of defined radiographic disease (Kellgren-Lawrence grade ≥2), is associated with a significant burden of disability in walking, stair climbing, mobility, and everyday housekeeping duties in farming communities in northern rural China. People aged 50 years and older with knee pain were more than twice as likely to report difficulty with these activities and tasks compared with people without knee pain, even when the results were adjusted for age, BMI, gender, formal education, and the presence of back pain. Furthermore, among the large proportion of people in this community reporting knee pain in the past 12 months (50%), the concomitant presence of radiographic disease (Kellgren-Lawrence grade ≥2) was associated with increased odds of reporting episodes of unbearable pain, restricted activity due to knee pain, and a greater use of analgesics or various health services for knee pain. Radiographic disease severity was clearly positively linked to symptoms.

The high prevalence of knee pain and knee OA in this farming community is probably not unexpected, even with the low prevalence of obesity. Heavy occupational activity is a well-established risk factor for incident knee OA [18,19]. As expected, symptomatic knee OA was associated with aging, BMI, and being a female person. Those with knee OA were less likely to be farmers or still working, a finding that can probably partly be attributed to the higher proportion of female subjects in this group (68%) compared with those without radiographic disease (49%).

Interestingly, the SF-12 PCS was unable to detect self-reported physical disability in this cohort. The mean PCS score of 49.3 demonstrated for the most symptomatic group compares very favorably with U.S. population norms for people aged 45 to 54 and 55 to 64 years of 49.7 and 46.6, respectively. Clearly the SF-12 questionnaire was unable to detect the level of disability reported in this population, as evidenced by the consistent dose-response relation according to knee pain and OA status detected by using specifically measured usual daily activities (Table 2). The possible lack of sensitivity of the SF-12 PCS may be related to the queried physical activities not being specifically directed at lower-limb disability or a culturally associated unwillingness to admit to having "accomplished less than you would like" or that pain interfered with their working ability. A significant difference appeared in the SF-12 MCS. Possibly surprisingly, people with knee OA had higher SF-12 MCS compared with people without knee pain. However, this finding is partly driven by the higher age of the group with knee OA. A stronger positive correlation occurred between age and MCS (0.12) compared with age and PCS (0.01) in this cohort of people aged 50 years and older.

The pattern of use of medications and health services for knee pain in this rural Chinese community was both expected and surprising. The low use of total knee replacement surgery, despite high levels of pain, disability, and severe radiographic disease [13] was expected in this region because of both financial constraints and limited access in this region to elective orthopedic surgery. For patients without health insurance (> 95% of this rural population), a total knee replacement was estimated to cost 40,000 RMB (approximately $6,000 US or more [20]. However, the average annual income per person in this area was about 5,000 RMB [21]. In addition, the very high use of NSAIDs for knee pain (approximately 80%), was surprising both in absolute terms and relative to that of paracetamol (< 5%). This pattern of analgesia use should be of great concern, given the resultant high exposure to adverse medical events associated with long-term use of NSAIDs, particularly in older people [22]. For this reason, paracetamol is recommended as the first-line drug treatment in the management of OA knee pain, despite evidence that NSAIDs are mostly more efficacious than paracetamol [23]. As the price of both forms of analgesia is fairly equivalent, the question arises as to why the villagers are focusing on using NSAIDs and ignoring paracetamol for their knee pain? Paracetamol may be perceived as not being able to provide sufficient pain relief to continue the heavy physical occupational activity required in rural regions of developing countries. However, the limited use of paracetamol reported indicates that paracetamol is not even being used during periods of less-severe knee pain or reduced occupational demands.

Several characteristics of the Wuchuan OA Study are worth noting. First, a rigorous sampling strategy was used, and the response rate was excellent (91%). Importantly, survey participants reporting knee pain were unaware of their knee OA status when completing the questionnaires evaluating physical function and disability. However, radiographic disease case definition was restricted to the tibiofemoral joints, so the knee OA prevalence estimates presented are conservative, as the patellofemoral joint was not included. The absence of information on the patellofemoral joint may also help explain the low percentage of those with knee pain who have radiographic OA, a lower percentage than found in other studies [4]. The prevalence of knee pain was higher than has been seen elsewhere [24] and suggests that the burden of knee pain and associated health care use is greater in this community than in other communities, especially those from urban or developed environments.

The disability and use of health services demonstrated in this survey can be generalized only to similar farming communities in Northern China, not to the population of Inner Mongolia in general. Participants in the Wuchuan OA Study were almost exclusively Han Chinese (99%), whereas this ethnic group makes up only 79% of the Inner Mongolian population, Mongolians accounting for most of the remaining 21%. The lifestyles of these two ethnic groups are very different; therefore, the prevalence of knee OA disability and patterns of medication and health services use may also be very dissimilar.

## Conclusions

Given the high prevalence of knee pain in Wuchuan with an associated increased physical disability, NSAIDs, and health services use, we suggest that knee pain and symptomatic knee OA represent a major public health concern in rural China. With the availability of knee-replacement surgery severely limited and occupational demands persisting into middle and older years, knee OA will continue to be a major source of disability among Chinese adults in rural areas, where most Chinese still live. Access to more sophisticated farming equipment to reduce the heavy physical demands of farming or timely availability of knee-replacement surgery may be cost-effective measures to reduce this burden of pain and disability and possible NSAIDs-related comorbidity.

## Abbreviations

BMI: body mass index; NSAIDs: nonsteroidal antiinflammatory drugs; OA: osteoarthritis; SF-12 MCS: Short-form 12 mental component summary score; SF-12 PCS: Short-form 12 physical component summary score.

## Competing interests

The authors declare that they have no competing interests.

## Authors' contributions

JL participated in the concept and design of the study, and the acquisition and interpretation of the data. MF participated in the concept and design of the study, data interpretation, and helped draft the manuscript. XK participated in the concept and design of the study, data acquisition, and helped draft the manuscript. HL participated in the concept and design of the study, data interpretation, and helped draft the manuscript. YK participated in the concept and design of the study and data acquisition and interpretation. ZW participated in the concept and design of the study. YZ participated in the concept and design of the study, data acquisition, and helped draft the manuscript. SS performed the statistical analysis. All authors read and approved the final manuscript.
